# Entering the 27% Era: Practical Design Rules for Single-Junction Perovskite Solar Cells

**DOI:** 10.1021/acsenergylett.6c00205

**Published:** 2026-02-25

**Authors:** Luigi Angelo Castriotta

**Affiliations:** CHOSE (Centre for Hybrid and Organic Solar Energy), Department of Electronic Engineering, 9318 University of Rome Tor Vergata, Via del Politecnico 1, 00133 Rome, Italy

Recent reports of single-junction perovskite solar cells exceeding 27% power conversion efficiency (PCE) mark a consolidation phase in device physics and materials engineering, reflecting convergence in materials choice and device design. Across independent demonstrations reported between late 2025 and early 2026, the perovskite composition, deposition routes, and device architectures are strikingly similar. This convergence indicates that further efficiency gains are now dictated by how precisely ionic species, interfaces, and contacts are controlled, particularly under operational conditions. This Viewpoint examines representative >27% single-junction perovskite solar cells and distills the practical design rules that underpin both their record efficiencies and their simultaneously improved operational stability. The discussion is framed to focus on quantitative performance metrics, interfacial physics, and mechanisms relevant to operational stability rather than record-centric narratives.

## From “Highly Efficient” to Quantitatively Justified Performance

The perovskite research community has repeatedly been cautioned against the uncritical use of nonquantifiable descriptors such as “highly efficient” and “stable”.^7^ The transition into the 27% efficiency regime provides a useful stress test for this principle: what, specifically, has changed compared to the already mature ∼26% class? The answer is not a new perovskite composition, nor a radical device architecture. Instead, the difference emerges from the systematic elimination of interfacial losses that were previously tolerated. At efficiencies approaching the radiative limit, small deviations in band alignment, molecular packing, and ionic redistribution translate directly into measurable voltage and fill-factor losses. Consequently, the 27% threshold should be viewed less as a headline milestone and more as evidence that the community has converged on a narrow set of physically justified design rules.

## Perovskite Composition: Controlled Chemistry Rather Than Novelty

All independently reported >27% devices rely on formamidinium-rich iodide perovskites with limited cesium incorporation, typically FA_0_._95_–_0_._97_Cs_0_._03_–_0_._05_PbI_3_, corresponding to a bandgap of ∼1.55 eV. Films are deposited by one-step spin coating from DMF/DMSO-based precursor solutions, followed by antisolvent dripping (typically chlorobenzene or isopropanol), or vacuum-assisted solvent extraction and thermal annealing. Chloride additives, most commonly MACl at 15–20 mol % relative to PbI_2_, remain essential to suppress nonperovskite phases and promote uniform crystallization. What differentiates the current >27% devices from earlier high-efficiency cells is the explicit management of halide chemistry during film formation. Rather than relying primarily on postdeposition passivation, recent demonstrations exert control over chloride retention and vertical distribution during crystallization, thereby suppressing halide segregation that would otherwise induce interfacial band bending and enhanced recombination. In parallel, precursor conditioning strategies that stabilize iodide redox chemistry reduce the formation of iodine vacancies and Pb-related deep traps prior to crystallization. Specific methods include: (i) optimizing MACl concentration (15–20 mol %) and DMSO:DMF ratios to control crystallization kinetics and chloride incorporation, (ii) tuning antisolvent timing and volume to regulate nucleation density and halide distribution, and (iii) controlled thermal ramping rates during annealing to manage chloride evaporation and prevent gradient formation. These strategies suppress halide segregation that would otherwise induce interfacial band bending and enhanced recombination. Possible pathways to improve film formation control include: real-time monitoring of halide distribution during crystallization using in situ spectroscopic techniques, computational prediction of additive-halide interaction energetics to guide precursor design, and vapor-phase halide delivery methods that decouple nucleation from compositional control. Emerging approaches such as sequential halide incorporation through multistep solution processing or halide-exchange post-treatments may enable independent optimization of bulk composition and interfacial stoichiometry, addressing the current limitation that single-step deposition couples these parameters.

Defect suppression for this class of devices is, therefore, shifted upstream, from surface treatments to precursor chemistry; precursor conditioning reduces bulk defect density, while postdeposition surface treatments address interfacial trap states, reflecting a more mature understanding of defect energetics.^1,2,4^

## Device Architecture: Why Inverted p–i–n Stacks Dominate above 27%

All reported >27% single-junction devices converge on inverted (p–i–n) architectures, typically ITO (or FTO)/SAM/perovskite/C_60_(or PCBM)/BCP/Ag (see Figure 1). This dominance is not a coincidence.^8^ Inverted architectures enable molecular-level control of both buried and top interfaces while minimizing hysteresis and allowing electrostatic dipoles and ion-blocking functionalities to be introduced without perturbing bulk transport. On the hole-selective side, carbazole-based phosphonic-acid self-assembled monolayers (SAMs) have become universal.^9^ The function of these SAMs extends well beyond energy-level alignment. Dense molecular packing, strong anchoring to oxide substrates, and controlled dipole formation suppress interfacial recombination and reduce local shunting pathways. Recent strategies that enhance Coulombic interactions between anchoring groups and the underlying TCO or NiO_x_ produce compact, chemically robust monolayers, enabling certified efficiencies above 27%. Electron-selective contacts are exclusively based fullerene-based materials, C_60_ or PCBM with thin bathocuproine (BCP) interlayers. This combination allows precise tuning of interfacial energetics and provides a platform for introducing molecular interlayers that simultaneously improve electronic selectivity and ionic stability.^6^ Despite this convergence on p-i-n stacks, small variations exist across the six >27% devices. Substrate choices include ITO (refs 1, 3, 6 ) versus FTO with or without NiO_x_ interlayers (refs 2, 4, 5 ), affecting work function alignment and optical transmission. SAM chemistry varies, Me-4PACz dominates (refs 2, 4, 6 ), but 4PADCB (ref 1) and CbzNaph (ref 3) offer different dipole moments, while SAM modifications with LiOH (ref 6) or Al_2_O_3_ underlayers (ref 1) enhance anchoring stability. Electron contacts show similar diversity: most use C_60_, but ref 2 employs C_60_/SnO_2_ bilayers and ref 5 uses PCBM with Cu electrodes instead of Ag. In perovskite/C_60_ interlayers, PEABr (refs 1, 3, 6 ), PDI_2_ (ref 4), PI (ref 1), and porphyrin derivatives (ref 5) are used, each targeting different loss mechanisms (surface traps, electron extraction barriers, crystallographic orientation, or ion migration). These variations indicate multiple pathways to >27%, with optimal stack choice depending on which interfacial loss dominates in a given processing environment.

**Figure 1:**
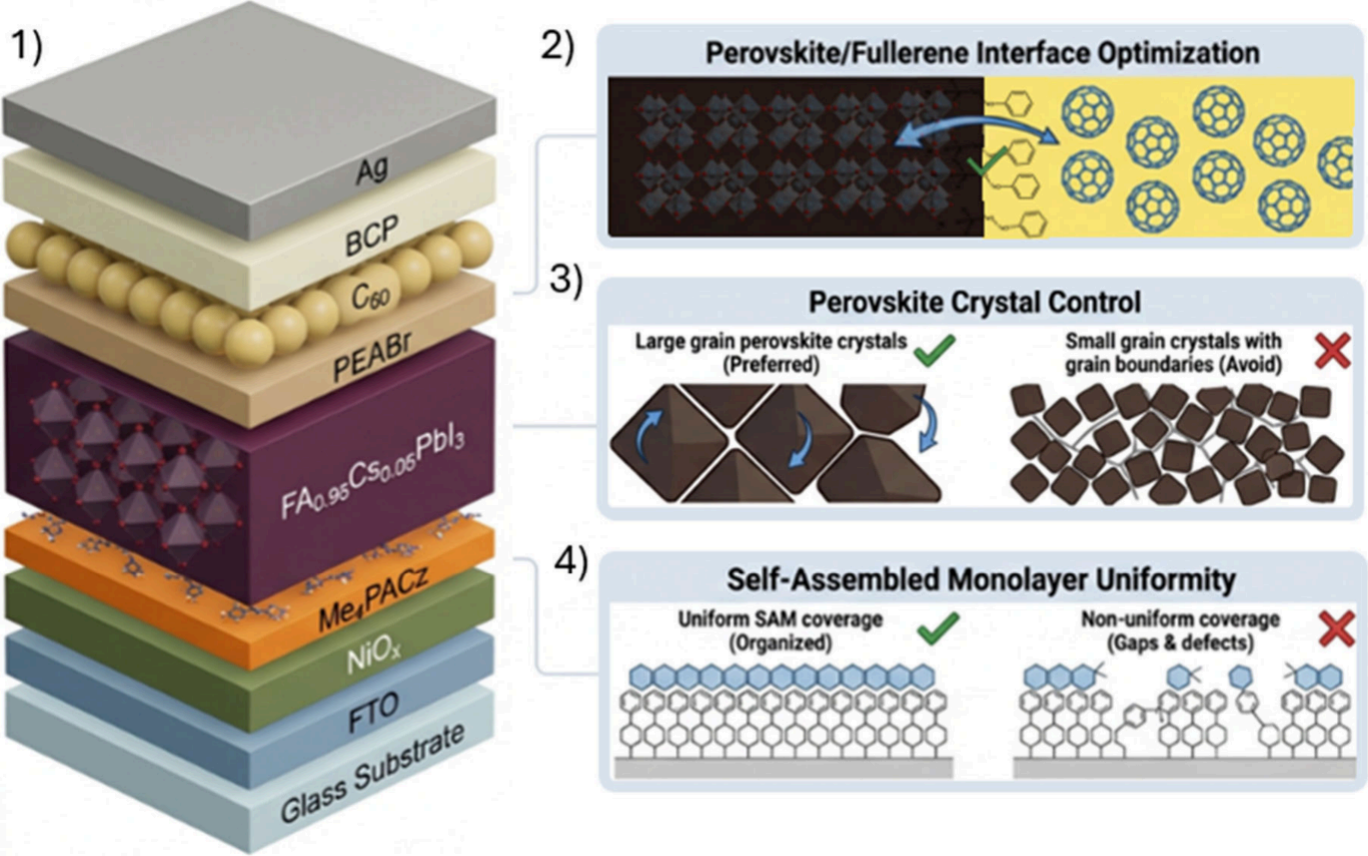
Practical design rules for getting >27% perovskite solar cells. 1) Inverted structure baseline structure composed by Glass/FTO/NiOx/Me_4_PACz/FA_0.95_Cs_0.05_PbI_3_/PEABr/C_60_/BCP/Ag, 2) Perovskite/fullerene improved interface, 3) Perovskite crystal control and 4) Self-assembled monolayer uniformity strategies.

## Stability as an Interface-Integrated Design Parameter

A defining feature of the >27% efficiency class is that efficiency gains are accompanied by demonstrable improvements in operational stability. Importantly, this stability is not achieved through encapsulation alone, but through deliberate suppression of ion migration at critical interfaces. At the perovskite/C_60_ interface, small-molecule interlayers are introduced to increase defect formation energies at the perovskite surface and promote denser fullerene packing. These interlayers generate favorable interfacial dipoles, reduce nonradiative recombination, and act as effective barriers against ion diffusion under illumination and thermal stress. Devices employing these strategies routinely retain >95% of their initial PCE after 1,000–1,500 h of maximum power point tracking under one-sun illumination and show stable operation at elevated temperatures (≈85 °C). The >27% class demonstrates that when interfacial ion dynamics are addressed at the molecular level, efficiency and stability improvements are not mutually exclusive.^3^

## Why the Difference Emerges above 27%

The step from ∼26% to >27% efficiency is driven primarily by the coordinated optimization of interfaces rather than by improvements in bulk transport. On the hole-selective side, compact and chemically robust SAMs suppress interfacial recombination by ensuring complete molecular coverage and minimizing energetic offsets. Dipole-engineered carbazole SAMs reduce valence-band offsets to below 0.1 eV while stabilizing favorable perovskite orientations at the buried interface.^3,5^ On the electron-selective side, ion-shielding interlayers transform C_60_ from a passive electron transporter into an active barrier against ionic redistribution. This focus on suppressing nonradiative losses at interfaces is consistent with analyses showing that efficiency gains in single-junction perovskite solar cells are increasingly limited by residual voltage losses as devices approach their radiative efficiency limits.^10^ By increasing defect formation energies and suppressing ion accumulation under bias, these interfaces stabilize both voltage and fill factor under prolonged operation.^3^ Analysis of certified performance metrics (Table 1) reveals that while interfacial passivation strategies target recombination losses, the >27% threshold is reached through different pathways across the six studies. Refs 1, 4 , and^5^ achieve >27% primarily through Jsc improvements (26.5–26.7 mA/cm^2^) rather than V_oc_ gains, with V_oc_ remaining at 1.188–1.192 V. In contrast, refs 2 and 6 show V_oc_ approaching 1.198 V with more modest J_sc_ (26.2–26.4 mA/cm^2^). This divergence indicates that interfacial engineering affects multiple loss mechanisms simultaneously: improved perovskite crystallization and surface planarization enhance optical absorption and charge collection (increasing J_sc_), while reduced interfacial trap densities suppress recombination (increasing V_oc_). The relative contribution of each pathway depends on which loss mechanism was dominant in the baseline device. Devices with already-optimized V_oc_ (∼1.18 V) gain efficiency through J_sc_ and FF improvements enabled by better interfacial contact uniformity, while devices with residual recombination losses show V_oc_ gains. The >27% class thus represents optimization convergence where all loss pathways, optical, recombination, and resistive, are simultaneously minimized. Table 2 summarizes the design rules to obtain such recombination loss reduction.

**Table 1:** Highlight the Key Architectural, Performance, and Stability Features of Single-Junction Perovskite Solar Cells Exceeding 27% Efficiency^a^

**Architecture**	**Key Strategy**	**PCE (Certified) (%)**	**Area (cm** ^**2**^ **)**	**Stability (Headline)**	**Ref (Date)**
**Glass/ITO/4PADCB/Al**_**2**_ **O**_**3**_ **/FA**_**0.95**_ **Cs**_**0.05**_ **PbI**_**3**_ **/PI/PCBM/**BCP/Ag	Perovskite crystallization control	27.02 (26.88)	0.0536	T98.2 at 2000 h MPPT	^1^(2025/10)
**Glass/FTO/NiOx/Me-4PACz/FAPbI**_**3**_ **/3-Pych/3-MTPAI/C60/SnO**_**2**_ **/Ag/MgF**_**2**_	MACl perovskite control	27.2 (27.2)	0.074	T86.3 at 1529 h MPPT	^2^(2025/11)
**Glass/ITO/CbzNaph/Cs**_**0.05**_ **FA**_**0.9**_ **MA**_**0.05**_ **PbI**_**3**_ **/PEABr/SHF/C60/BCP/Ag**	Multistep interfacial passivation	27.02 (26.96)	0.0782	T100 at 1200 h MPPT	^3^(2025/11)
**Glass/FTO/NiOx/Me-4PACz/FA**_**0.95**_ **Cs**_**0.05**_ **PbI**_**3**_ **/PDI**_**2**_ **/C60/BCP/Ag**	Ion–defect dual management in perovskite	27.1 (27.1)	0.0535	T98.1 at 1200 h MPPT	^4^(2026/1)
**ARF/Glass/FTO/BrAs-PIE/Cs**_**0.05**_ **FA**_**0.88**_ **MA**_**0.07**_ **PbI**_**3**_ **/POEAI-NH**_**4**_ **SCN/tetrakis(pentafluorophenyl)porphyrin/PCBM/BCP/Cu**	Donor–acceptor interface engineering	27.28 (27.19)	0.0737	T93.7 1000 h, 85 °C, T95.5 at 1500 h MPPT	^5^(2026/1)
**Glass/ITO/NiOx/Me-4PACz-LiOH/Cs**_**0.05**_ **MA**_**0.05**_ **FA**_**0.9**_ **PbI**_**3**_ **/PEABr/PCBM-C60/BCP/Ag/MgF**_**2**_	Coulomb-stabilized SAM	27.3 (27.32)	0.0535	T93 at 2000 h at 65–85 °C, MPPT	^6^(preprint 2026/1)

a Full fabrication protocols, material compositions, processing conditions, and vendor information are provided in Table S1 in the Supporting Information, updated up to Feb 24th, 2026.

**Table 2:** List of Design Rules to Achieve >27% Perovskite Solar Cells Efficiency

**Design Rule**	**Description**	**Reason**	**Target**
**1. Perovskite Composition**	FA_0_._95–0_._97_Cs_0_._03–0_._05_PbI_3_; Bandgap ∼ 1.55 eV; MACl additive 15–20 mol % relative to PbI_2_	Optimal balance between efficiency and phase stability; suppresses nonperovskite phases and promotes uniform crystallization	Minimize deep-trap density; suppress halide segregation; achieve uniform film crystallization
**2. Perovskite Deposition Protocol**	One-step spin coating from DMF/DMSO; vacuum-assisted solvent extraction; controlled thermal annealing	Enables precise control of chloride retention and vertical distribution during crystallization	Manage chloride distribution to prevent interfacial band bending; stabilize iodide redox chemistry
**3. Device Architecture**	Inverted p-i-n configuration: Glass/ITO(or FTO)/SAM/Perovskite/C_60_/BCP/Ag	Enables molecular-level control of buried and top interfaces; minimizes hysteresis; allows introduction of ion-blocking functionalities	Suppress hysteresis (hysteresis index <5%); enable precise interfacial energetics control
**4. Hole-Selective Contact**	Carbazole-based phosphonic-acid SAMs (e.g., Me-4PACz, 4PADCB, CbzNaph) with enhanced anchoring	Dense molecular packing and strong anchoring suppress interfacial recombination and local shunting pathways	Valence-band offset <0.1 eV; complete molecular coverage; minimize interfacial recombination velocity
**5. Sam Enhancement Strategy**	Coulombic stabilization through compositional modifications (e.g., LiOH treatment, Al_2_O_3_ interlayer)	Increases interaction between anchoring groups and TCO/NiO_x_; produces chemically robust, compact monolayers	Enhance SAM compactness and uniformity; improve long-term chemical stability
**6. Electron-Selective Contact**	C_60_ or PCBM + BCP interlayer (<10 nm) + Ag electrode	Precise tuning of interfacial energetics; provides platform for molecular interlayer integration	Optimize electron extraction efficiency; minimize electron-extraction losses
**7. Perovskite/C**_**60**_ **Interlayer**	Small-molecule passivation layers (e.g., PDI_2_, PI, PEABr, porphyrin derivatives, donor–acceptor systems)	Increases defect formation energies at perovskite surface; promotes denser fullerene packing; generates favorable interfacial dipoles; acts as ion-diffusion barrier	Suppress ion accumulation under bias; reduce nonradiative recombination at top interface

## Outlook

The emergence of single-junction perovskite solar cells exceeding 27% efficiency reflects a maturation of design rules. Standardizing formamidinium-rich perovskites near 1.55 eV, adopting inverted p–i–n architectures, and enforcing strict control over halide distribution, redox chemistry, and interfacial ion dynamics have established a clear route to performance over 27%. The same design choices that suppress nonradiative recombination also mitigate degradation pathways, resolving the trade-off between efficiency and stability.

Further efficiency gains will arise from incremental reductions in interfacial losses. The design rules from the >27% class provide a framework for upscaling and durability, aligning laboratory achievements with practical deployment. The question is how consistently these design rules translate to larger device areas and extended operational lifetimes. The 27% milestone represents a transition from exploratory optimization to disciplined, quantitatively justified device engineering.

## Supplementary Material



## Notes

Full fabrication protocols, material compositions, processing conditions, and vendor information are provided in Table S1, updated up to Feb 24, 2026, together with a Standard Operating Procedure summarizing the most common materials and fabrication steps to follow to get highly efficient devices. (PDF)
