# Supplementary material for: Entering the 27% Era: Practical Design Rules for Single-Junction Perovskite Solar Cells
Source: ACS Energy Lett. 2026 Feb 25;11(3):2378–81. doi: 10.1021/acsenergylett.6c00205 (PMC12993994; doi:10.1021/acsenergylett.6c00205)
Supplement: Supplementary file 1 [file nz6c00205_si_001.pdf]

Supporting Information

Entering the 27% Era: Practical Design Rules for Single-Junction Perovskite Solar Cells

Luigi Angelo Castriotta[1*]

1. CHOSE (Centre for Hybrid and Organic Solar Energy), Department of Electronic Engineering, University of Rome Tor Vergata, Via del Politecnico 1, 00133 Rome, Italy

Table 1. Fabrication Protocol details of Perovskite Solar Cells >27% PCE, updated up to Jan 22nd, 2026.

	Architecture and process details	*Vendors*	*PCE/Area*	*Stability*
**Ref [1]**	**Glass/ITO/4PADCB/Al_2O_3/$FA_{0.95}Cs_{0.05}PbI_3$/PI/ PCBM/BCP/Ag**			
Substrate	The ITO glass substrate was ultrasonically cleaned with detergent solution, deionized water, ethanol and IPA, respectively, and then dried using a high-pressure N_2 gas flow. The dried ITO glass substrate was treated with UV-ozone and then transferred to a glovebox to deposit the functional layer.	Not declared	PCE of 27.02% (certified 26.88%), 0.0536cm^2; aperture area of 11.09 cm^2 and all-perovskite tandem solar cells achieve a certified steady-state efficiency of 23.18% and a certified PCE of 29.07%, respectively.	T98.2 after 2,000 h under continuous illumination. Damp heat (85 °C and 85% relative humidity (RH)) in an ageing chamber following IEC 61215:2016, T95.9 after 600 h.
HTL	The self-assembled molecule (SAM) layer was prepared via spin-coating 4PADCB solution (0.5 mg ml−1 in ethanol) onto the ITO glass substrate at 3,000 revolutions per min (r.p.m.) for 30 s and then annealing at 100 °C for 10 min.	Not declared		
	The Al_2O_3 nanoplate dispersions in IPA were spin-coated onto the SAM layer.	Sigma Aldrich		
Perovskite + Passivators	The 1.5 M $FA_{0.95}Cs_{0.05}PbI_3$ perovskite precursor solution was prepared by dissolving PbI_2 (1.53 mmol), FAI (1.425 mmol), CsI (0.075 mmol) and MACl (0.075 mmol) in a mixed solvent of DMF:DMSO (volume ratio, 5:1). For the additive-based perovskite, the PHNS and BNAC molecules were mixed and dissolved in DMSO and added to the perovskite precursor at molar ratios of 1%. The perovskite film was prepared by spin-coating the perovskite precursor solution at 4,000 r.p.m. for 50 s. CB as an antisolvent was injected into the centre of the rotating substrate at 20 s before the end of the spin programme. The perovskite films were annealed at 100 °C for 30 min. PI solution was spin-coated on top of the perovskite layer at 3,000 r.p.m. for 30 s and annealed at 100 °C for 5 min.	DMF, DMSO, CB, Sigma Aldrich; PbI2, FAI, Advanced Election Technology; CsI, MACl, PHNS, PI, TCI; BNAC, Macklin.		
ETL	The PCBM film was prepared via spin-coating the PCBM solution (20 mg ml^{-1} in CB) onto the perovskite film at 3,000 r.p.m. for 30 s and then annealing at 70 °C for 10 min.	Advanced Election Technology		
	The BCP film was prepared by spin-coating a BCP saturated solution in IPA onto the PCBM film at 5,000 r.p.m. for 30 s and then annealing at 70 °C for 10 min.	TCI		
Top Electrode	120-nm-thick layer of Ag was deposited onto the BCP film via thermal evaporation in a high vacuum.	Not declared		
**Ref [2]**	**Glass/FTO/NiOx/Me-4PACz/$FAPbI_3$/3-Pych/3-MTPAI/C60/SnO_2/Ag/MgF_2**	*Vendors*	*PCE/Area*	*Stability*
Substrate	FTO cleaned by sequentially washing with detergent, deionized water, acetone, and isopropanol (IPA). Before use, the FTO was cleaned with ultraviolet ozone for 30 min.	Not declared	A certified steady-state power conversion efficiency (PCE) of 27.2% (device area and measured mask area: 0.108cm^2 and 0.074cm^2, respectively).	Devices retained 86.3% of their initial PCE after 1529 hours of continuous maximum power point tracking (MPPT) under 1 Sun condition
HTL	NiOx, substrate spin-coated with a thin layer of NiOx nanoparticle film (10 mg/ml NiOx water solution with 40 mol% H_2O_2 addition) at 1500 rpm for 30 s, annealed in ambient air at 150 °C for 10min.	Advanced Election Technology		
	0.5 mg/ml Me-4PACz was deposited on the NiOx at 4000 rpm for 30s and annealed at 120 °C for 10 min.	TCI		
Perovskite + Passivators	FAI and PbI_2 with additional 6.5 mol% PbI_2 and 19.5 mol% MACl (the molar ratio is 1:3) in mixture solvents of DMF and NMP (830:170 v/v). Besides, FAfa (0.65 mg/mL) was added into the precursor to increase the crystallinity. For the homogenized vertical chlorine distribution (HVCD) perovskite, PB powder (1.5 mg/mL) can be either dissolved in the precursor solution directly or pre-dissolved in the DMF to obtain better	DMF, NMP, PbI_2, Sigma Aldrich; FAI, MACl, 3-(methylthio)propylamine hydrochloride (3-MTPAI), Xi'an Polymer Light		

	dispersibility. The precursors were stirring at room temperature for about 2h. Finally, the precursor solutions were filtered by 0.22 μm polytetrafluoroethylene filters before use. 1.8M perovskite deposited on the FTO/NiOx/Me-4PACz substrate at 4000 rpm for 7 s followed by vacuum flashing in the transfer chamber of the glove box for 1 min. The precursor film was annealed in N_2 at 105 °C for 40 min to remove the rest solvents. After that, 0.5 mg/ml 3-Pych and 2.0 mg/mL 3-MTPAI were sequentially coated on the perovskite surface at 5000 rpm for 20 s and annealed at 100 °C for 5 min.	Technology; Formimidamide formate (FAfa), Aladdin; Potassium binoxalate (PB) and 3-Pyridinecarboxamidine hydrochloride (3-Pych), Adamas		
ETL	15 nm C60 deposited via thermal evaporation.	Nano-C		
	Atomic layer deposition chamber for SnO_2 layer deposition	Not declared		
Top Electrode + ARC	150 nm thickness of Ag thermally evaporated, MgF_2 has been adopted as anti-reflection layer on the glass side by thermal evaporation method with the thickness about 160 nm.	Not declared		
**Ref [3]**	**Glass/ITO/CbzNaph/$Cs_{0.05}FA_{0.9}MA_{0.05}PbI_3$/PEABr/SHF/C60/BCP/Ag**	*Vendors*	*PCE/Area*	*Stability*
Substrate	Cleaned with deionized water, acetone and isopropyl alcohol in this order for 15 min. The substrates were further exposed to ultraviolet ozone for 30 min.	Not declared		
HTL	CbzNaph (0.3 mg ml−1) was dissolved in absolute ethanol and used as a hole-selective contact. The SAM solution was spin coated on the substrate at 3,000 rpm in a two-step process with 3-s acceleration and then kept for 30 s. Then,<br>the substrate–SAM was transferred onto a hotplate and annealed at 100 °C for 10 min in a N2-filled glovebox.	Luminescence Technology		
Perovskite + Passivators	1.55 M of $Cs_{0.05}FA_{0.9}MA_{0.05}PbI_3$ with 3% excess PbI_2 in dimethylformamide/dimethyl sulfoxide with a volume ratio of 4:1. Then, 10 mg ml−1 of methylammonium chloride was added to the precursor solution to improve the film morphology. The mixture was stirred overnight and then filtered through a 0.22-μm polytetrafluoroethylene membrane before use. The precursor solution was spin coated at 1,000 rpm for 10 s, followed by 5,000 rpm for 40 s. In the last 5 s during the spin-coating procedure, 200 μl of chlorobenzene as the antisolvent was dropped onto the substrate. The precast films were then annealed at 100 °C for 30 min to obtain perovskite films. For initial surface passivation, 1 mg ml−1 of phenethylammonium bromide was subsequently used, dissolved in isopropyl alcohol/dimethyl sulfoxide (195:5, v/v), which was spin coated on the prepared perovskite films at 5,000 rpm for 30 s and then annealed at 100 °C for 10 min. SHF was dissolved in isopropyl alcohol<br>(in varying concentrations of 3 mM) and spin coated onto the film surface, under 3,000 rpm for 30 s at a ramp of 1,000 rpm s−1 and then annealed at 100 °C for 5 min.	FAI (99.99%), MAI (99.99%), MACl (99.99%), Dyenamo; PbI2, TCI; CsI, (99.999%, AB 109298), abcr Gute Chemie; PEABr, Greatcell Solar Materials; DMSO, DMF, IPA, EtOH, CB, Sigma Aldrich; SHF (C4F7NaO2), Santa Cruz Biotechnology.	27.02% (certified 26.96% with a maximum-power-point-tracking PCE of 26.61%), accredited by Fujian Metrology Institute (National PV Industry Measurement and Testing Center), 0.0782cm^2 area.	Perovskite solar cells retain 100% of their initial efficiency following 1,200 h of continuous 1-sun illumination at the maximum power point. Thermal stability, retaining 92% of the initial PCE when ageing at 85 °C for 1,800 h and 94% after 200 thermal cycles between –40 °C and +85 °C.
ETL	23 nm C60 deposited via thermal evaporation by temperature control, under 1.0 × 10−6 mbar.	(≥99.99%), CreaPhys		
	7 nm BCP deposited via thermal evaporation by temperature control, under 1.0 × 10−6 mbar.	(99.8%), Ossila		
Top Electrode	100 nm Ag deposited via thermal evaporation by power control, under 1.0 × 10−6 mbar.	shots (2–3 mm, 99.999%), Alfa Aesar		
**Ref [4]**	**Glass/FTO/NiOx/Me-4PACz/$FA_{0.95}Cs_{0.05}PbI_3$/PDI_2/C60/BCP/Ag**	*Vendors*	*PCE/Area*	*Stability*
Substrate	FTO glass substrates were cleaned with a detergent solution, deionized water, acetone, and anhydrous ethanol for 10 min, respectively. Next, the substrates were further cleaned with plasma treatment for 15 min.	Not declared	Certified steady-state efficiency of 27.10%, The effective area of the mask is 0.0535 cm^2 with certification, Tianjin Institute of Metrological Supervision and Testing	Unencapsulated air with a relative humidity of 30±5% and a temperature of 25±5◦C with continuous LED irradiation, $T_{98.1}$ at 1200h.
HTL	NiOx films were fabricated by spin-coating the 10 mg/mL NiOx aqueous solution on the FTO substrates at 3000 rpm for 30 s, followed by annealing at 150 °C for 10 min.	Nickel (II) nitrate hexahydrate (Ni(NO3)2·6H2O), Aladdin for synthesizing the NiOx nanoparticles.		
	0.5 mg/mL Me-4PACz ethanol solution was spin-coated on NiOx film at 4000 rpm for 30 s, followed by annealing at 100 °C for 10 min.	Suzhou LiWei Tech Co., Ltd		

Perovskite + Passivators	1.8 M perovskite precursor solution was prepared through dissolving 228.7 mg FAI, 10 mg MACl, 18.2 mg CsI and 645.4 mg PbI_2 in a mixed solvent of DMF/DMSO (4:1, by volume). 0.5 mg N-Acetylsulfanilyl chloride (ABSC) was doped in the 1 mL perovskite precursor, spin-coated FTO/NiOx/Me-4PACz substrate for further crystallization using the vacuum flash evaporation method in air. The rapid pump-down is essential. Subsequently, the bare or ABSC-modulated perovskite film was annealed at 100 °C for 30 minutes in the air (with controlled humidity no more than 40%). Piperazine Dihydriodide (PDI_2, CAS:58464-47-4, with concentration of 0.4 mg/ml dissolved in IPA) was spin-coated on the crystallized perovskite film at 5000 rpm for 30 s, and annealed at 100 °C for 5 min.	FAI, 99.5%, Greatcell Solar Materials; PbI2, 99.99%, CsI, 99.9%, ChengDu Alfa Metal Material; DMF, DMSO, Alfa Aesar; Piperazine Dihydriodide (>98.0%(T)(N)),TCI. N-Acetylsulfanilyl chloride (ABSC, 98%), Macklin.		
ETL	30 nm C60 deposited via thermal evaporation, under a high vacuum of 5×10^{-4} Pa.	Advanced Election Technology		
	7 nm of BCP solution was evaporated on the C60 layer under a high vacuum 5×10^{-4} Pa.	Advanced Election Technology		
Top Electrode	120 nm Ag electrode is thermally evaporated.	Not declared		
**Ref [5]**	**ARF/Glass/FTO/BrAs-PIE/$Cs_{0.05}FA_{0.88}MA_{0.07}PbI_3$/POEAI-$NH_4SCN$/tetrakis(pentafluorophenyl)porphyrin/PCBM/BCP/Cu**	*Vendors*	*PCE/Area*	*Stability*
Substrate	An anti-reflective film (ARF, BA4076, Japan) was applied to the back of the FTO glass.	model number: HM-FTO-22 8 ohm/sq and a thickness of 2.2 mm, Beijing Huamin New Materials Technology Co., Ltd	27.28% (27.19% certified), with a maximum-power-point-tracking certified PCE of 26.85%, accredited by Fujian Metrology Institute (National PV Industry Measurement and Testing Center), $0.0737cm^2$ area.	$T_{93.7}$ 1,000 h at 85 °C, $T_{95.5}$ 1,500 h of illumination at MPP (65 °C), ISOS-L-2 standard Protocol.
HTL	A 1 mM solution of BrAs in methanol was spin coated onto a cleaned and ultraviolet-treated FTO substrate at 2,000 rpm for 30 s and annealed at 100 °C for 10 min.	Own Synthetised		
	PIE solutions with isopropanol as the solvent were spin coated at 5,000 rpm for 30 s and annealed at 100 °C for 10 min.	PIE, Shanghai Aladdin Biochemical Technology		
Perovskite + Passivators	A 1.4-M $Cs_{0.05}FA_{0.88}MA_{0.07}PbI3$ precursor solution in DMF:DMSO (4:1) was spin coated onto FTO/SAM at 2,000 rpm for 35 s, followed by an increase to 6,000 rpm for 10 s. Isopropanol was introduced as an antisolvent 8 s before the end, and the film was annealed at 100 °C for 15 min. A 1 mg ml−1 solution of phenoxyethylammonium iodide (POEAI) in isopropanol:DMF (100:1) with 0.25 mg of NH_4SCN was spin coated at 5,000 rpm and annealed at 100 °C for 10 min. A 2 mg ml−1 solution of tetrakis(pentafluorophenyl)porphyrin in chlorobenzene with 1 mg of polystyrene derivative was then spin coated at 5,000 rpm.	Formamidine iodide (>99.5%), Greatcell Solar Materials. CsI (>99.99%), FACl (>99.99%) MACl (>99.5%), Xi'an Polymer Light Technology. PbI2 (>99.99%), Xi'an E-Light New Material. DMF (99.8%), DMSO (99.8%) and methanol (99.9%), Sigma Aldrich.		
ETL	20 mg ml−1 solution of [6,6]-phenyl-C61-butyric acid methyl ester in chlorobenzene:trichloromethane (1:1) spin coated at 4,000 rpm for 30 s	Lumtec		
	spin coating of bathocuproine at 5,000 rpm for 30 s.	Not declared		
Top Electrode	120-nm Cu back electrode.	Not declared		
**Ref [6]**	**Glass/ITO/NiOx/Me-4PACz-LiOH/$Cs_{0.05}MA_{0.05}FA_{0.9}PbI_3$/PEABr/PCBM-C60/BCP/Ag/$MgF_2$**	*Vendors*	*PCE/Area*	*Stability*
Substrate	ITO glasses were continuously washed in an ultrasonic bath for 15 min in detergent-deionized water solution, acetone and ethanol, respectively. The ITO glasses were dried with N_2 and then treated with UV ozone for 7 min to increase hydrophilicity.	Not declared	Certified steady-state efficiency of 27.32%, certified by	Devices maintained 94.5% and 93.3% of their initial efficiencies after

HTL	NiOx-NPs (20 mg ml−1 in H2O) was deposited on ITO at 2000 rpm for 30 s and then heated at 100 °C for 10 min. The coated ITO was then moved to a glove box.	Nickel nitrate hexahydrate (Ni(NO3)2·6H2O, 99.999%), Sigma Aldrich. Nickel oxide nanoparticles (NiOx-NPs) were synthesized according to a previous literature	Tianjin Institute of Metrological Supervision and Testing, 0.0535 cm^2 mask area.	continuous operation under solar light exposure for 2,000 hours at 65°C and 1,000 hours at 85°C (ISOS-L-3 protocol), respectively. In addition, 94.6% of initial efficiencies were retained in a device after 1,600 hours of heat treatment at 85°C (ISOS D-2I).
	The LiOH-reacted Me-4PACz were fabricated by spin-coating the Me-4PACz (0.3 mg mL−1 in ethanol) and LiOH (molar ratio=1:0.5) on NiOx-NPs substrates at 4000 rpm for 30 s, followed by thermal annealing at 100 °C for 10 min. After that, the substrates needed to be washed with ethanol through spin-coating at 4000 rpm for 30 s, then annealing at 100 °C for 5 min.	Lithium hydroxide (LiOH, 99.99%), Aladdin; Me-4PACz, TCI.		
Perovskite + Passivators	1.7 M $Cs_{0.05}MA_{0.05}FA_{0.9}PbI_3$ perovskite precursors were prepared by dissolving 0.085 mmol CsI, 0.085 mmol MAI, 1.53 mmol FAI, and 1.7 mmol PbI2 in DMF: DMSO (4:1 volume ratio, v: v), 5% MAPbCl3 excess were added to improve the quality of perovskite films. The precursor solutions were stirred at 60ºC for 1 h and then filtered using a 0.22 μm polytetrafluoroethylene membrane before use. The perovskite solution was spin coated on the Me-4PACz or LiOH-reacted Me4PACz according to procedure that was increased from 1000 rpm for 10 s to 5000 rpm for 30 s, dropping 150 μL chlorobenzene antisolvent for 10 s before the end of the procedure. Then heated at 110 °C for 10 min to obtain the bright perovskite film. PEABr with concentration of 1 mg/ml in IPA and DMSO solution (volume ratio 200:1) was dynamically spin-coated on top (4000 rpm, 30 s) and annealed at 100 °C for 5 min.	CB (99.8%), DMSO (99.8%), DMF (99.8%), Sigma Aldrich; PbI_2, CsI, MAI, $PbBr_2$, MACl, Xi'an Yuri Solar Co. Ltd; PEABr, FAI, Greatcell Solar Materials.		
ETL	PCBM and C60 mixed solution (25 mg mL−1, 4/1, w/w, 1mL CB), spin-coated at 3000 rpm for 30 s.	Not declared		
	BCP (0.5 mg mL−1 in IPA), spin coated on it at 5000 rpm for 30 s.	Not declared		
Top Electrode + ARC	thermally evaporating Ag (100 nm) electrodes (0.0535 cm^2 mask area). A 100-nm thick magnesium fluoride layer was deposited on the back of ITO substrate for transmittance enhancement.	Not declared		

STANDARD OPERATING PROCEDURE (SOP)

Glass/FTO/NiO_x/Me-4PACz/$Cs_{0.05}FA_{0.95}PbI_3$(+MACl)/PEABr/C_{60}/BCP/Ag

0. Materials, Solutions, and Equipment

Key chemicals:

- FTO-coated glass substrates
- NiO_x nanoparticle dispersion (or dry NPs for aqueous dispersion)
- Me-4PACz
- PbI_2, FAI, CsI, MACl
- PEABr
- DMF (anhydrous), DMSO (anhydrous), IPA (anhydrous), ethanol (absolute), chlorobenzene (CB)
- C_{60}, BCP, Ag pellets (evaporation grade)

Typical equipment:

- Ultrasonic bath, N_2 gun, plasma cleaner (or UV-ozone)
- Hotplates (100–150 °C), spin coater, micropipettes
- Glovebox (dry N_2 recommended) or controlled dry-air enclosure
- Thermal evaporator with thickness monitor

1. Substrate Preparation (Glass/FTO)

1. Label and mask the conductive side if needed.
2. Sequential ultrasonication (10 min each):
   a) detergent solution
   b) DI water
   c) acetone
   d) ethanol (or IPA)
3. Dry with N_2
4. Plasma treat 15 min immediately before NiO_x deposition.

2. NiO_x Hole Transport Layer (NP Film)

2.1 NiO_x solution

Target: 10 mg mL^{-1} NiO_x in DI water.
Example (10 mL): weigh 100 mg NiO_x NPs + add DI water to 10.0 mL.
Sonicate or vortex until uniform.

2.2 Deposition

1. Spin coat NiO_x: 3000 rpm, 30 s.
2. Anneal: 150 °C, 10 min.
3. Cool to room temperature.

3. Me-4PACz Self-Assembled Monolayer (SAM)

3.1 Me-4PACz solution

Target: 0.5 mg mL^{-1} Me-4PACz in absolute ethanol.
Example (10 mL): weigh 5.0 mg Me-4PACz + add ethanol to 10.0 mL. Mix until fully dissolved.

3.2 Deposition

1. Spin coat Me-4PACz: 4000 rpm, 30 s.
2. Anneal: 100 °C, 10 min.
3. Cool to room temperature before perovskite.

4. Perovskite Precursor Ink (Locked Baseline for Reproducibility)

Baseline composition: $Cs_{0.05}FA_{0.95}PbI_3$ with MACl additive.
Target concentration: 1.7 M.
Solvent: DMF:DMSO = 4:1 (v/v).
MACl additive: 20 mol% vs Pb (default for reproducibility).

4.1 Weighing recipe for 1.00 mL (1.7 M)

Solids to weigh:

- PbI_2: 783.7 mg
- FAI: 277.7 mg
- CsI: 22.1 mg
- MACl (20 mol% vs Pb): 22.9 mg

Solvent volumes (total 1.00 mL):

- DMF: 800 µL
- DMSO: 200 µL

4.2 Preparation

1. Add DMF and DMSO to a vial, then add solids.
2. Stir (or shake) at 60 °C for ~1 h until fully clear.
3. Warm-filter through 0.22 µm PTFE into a clean vial.
4. Use fresh same day; keep capped to avoid solvent loss and viscosity drift.

5. Perovskite Deposition (Antisolvent Method)

Environment: dry N_2 glovebox recommended (consistent low H_2O).
1. Spin program:
1) 1000 rpm, 10 s
2) 5000 rpm, 30 s
Antisolvent:
- Drip chlorobenzene (CB) 150 µL at 10 s before the end of step
2.Anneal: 110 °C, 10 min.

6. PEABr Surface Passivation (Locked Baseline)

6.1 PEABr solution

Target: 1.0 mg mL^{-1} PEABr in IPA:DMSO = 200:1 (v/v).
Example (2.00 mL): weigh 2.0 mg PEABr + add 2.00 mL IPA + add 10 µL DMSO; mix until clear.

6.2 Deposition

1. Dynamic spin coat: 4000 rpm, 30 s (dispense shortly after spin starts).
2. Anneal: 100 °C, 5 min.

7. Electron Transport Layer and Top Electrode (Thermal Evaporation)

Base pressure: ~5×10^{-4} Pa (or better).
Deposit sequentially:

- C_{60}: 30 nm
- BCP: 7 nm
- Ag: 120 nm (shadow mask defined active area)

8. Critical Control Points (for Batch-to-Batch Reproducibility)

1) Humidity control: keep perovskite deposition in consistently dry atmosphere (glovebox preferred).
2) Ink freshness: prepare perovskite ink fresh and keep capped; filter warm; discard if viscosity changes.
3) Antisolvent timing: keep drip at "end − 10 s" and fixed drip height/volume (same pipette each run).
4) Substrate/SAM: plasma immediately before NiO_x; avoid long delays between Me-4PACz and perovskite.
5) Hotplate accuracy: verify real surface temperature (110 °C and 100 °C steps).
6) Evaporation: keep identical thicknesses and base pressure; avoid long air exposure before C_{60}.

9. Safety and Waste

Pb salts (toxic), DMF (toxic), DMSO (skin permeation), chlorobenzene (harmful), IPA/EtOH (flammable).
Wear PPE and dispose Pb-containing waste according to institutional procedures.

References

(1) Zhou, Q.; Huang, G.; Wang, J.; Miao, T.; Chen, R.; Lei, X.; Xu, E.; Liu, S.; Zhu, H.; Tan, Z.; et al. Aromatic interaction-driven out-of-plane orientation for inverted perovskite solar cells with improved efficiency. *Nature Energy* **2025**, *10* (11), 1371-1381.

(2) Xiong, Z.; Zhang, Q.; Cai, K.; Zhou, H.; Song, Q.; Han, Z.; Kang, S.; Li, Y.; Jiang, Q.; Zhang, X.; et al. Homogenized chlorine distribution for >27% power conversion efficiency in perovskite solar cells. *Science* **2025**, *390* (6773), 638-642.

(3) Li, G.; Zhang, Z.; Agyei-Tuffour, B.; Wu, L.; Gries, T. W.; Prashanthan, K.; Musiienko, A.; Li, J.; Zhu, R.; Hart, L. J. F.; et al. Stabilizing high-efficiency perovskite solar cells via strategic interfacial contact engineering. *Nature Photonics* **2025**, *20* (1), 55-62.

(4) Lu, H.; Zhuang, X.; Ding, J.; Zhang, Z.; Li, M.; Li, C.; Wu, W.; Lu, M.; Liu, H.; Lin, Z.; et al. Ion-Defect Dual Management for Achieving Efficient Air-Processed Perovskite Solar Cells With Certified Efficiency 27.1%. *Advanced Materials* **2026***:* e17596.

(5) Tian, C.; Sun, A.; Chen, J.; Zhuang, R.; Chen, C.; Zheng, J.; Liu, S.; Du, J.; Chen, Q.; Lei, C.; et al. Photostable donor–acceptor interface for minimizing energy loss in inverted perovskite solar cells. *Nature Photonics* **2026**, 1-9.

(6) Yan, F.; Cao, Q.; Du, T.; Zhang, Z. Z.; Mei, J.; He, X.; Su, Z.; Feng, G.; Kang B.; Hou, J. et al. Improved compactness of self-assembled monolayers through Coulomb interaction enables highly efficient and stable perovskite solar cells. Research Square. 08 January **2026**, doi.org/10.21203/rs.3.rs-8070036/v1 (accessed 2026-02-17).
